# Direct Analysis in Real Time (DART) of an Organothiophosphate at Ultrahigh Resolution by Fourier Transform Ion Cyclotron Resonance Mass Spectrometry and Tandem Mass Spectrometry

**DOI:** 10.3390/ijms17010116

**Published:** 2016-01-16

**Authors:** Laszlo Prokai, Stanley M. Stevens

**Affiliations:** 1 Center for Neuroscience Discovery, University of North Texas Health Science Center, 3500 Camp Bowie Boulevard, Fort Worth, TX 76107, USA; smstevens@usf.edu; 2 Department of Cell Biology, Microbiology, and Molecular Biology, University of South Florida, 4202 E. Fowler Ave., Tampa, FL 33620, USA

**Keywords:** direct analysis in real time, mass spectrometry, Fourier transform ion cyclotron resonance, simulated chemical warfare agent, surface swabbing

## Abstract

Direct analysis in real time (DART) is a recently developed ambient ionization technique for mass spectrometry to enable rapid and sensitive analyses with little or no sample preparation. After swab-based field sampling, the organothiophosphate malathion was analyzed using DART-Fourier transform ion cyclotron resonance (FT-ICR) mass spectrometry (MS) and tandem mass spectrometry (MS/MS). Mass resolution was documented to be over 800,000 in full-scan MS mode and over 1,000,000 for an MS/MS product ion produced by collision-induced dissociation of the protonated analyte. Mass measurement accuracy below 1 ppm was obtained for all DART-generated ions that belonged to the test compound in the mass spectra acquired using only external mass calibration. This high mass measurement accuracy, achievable at present only through FTMS, was required for unequivocal identification of the corresponding molecular formulae.

## 1. Introduction

Direct analysis in real time (DART) is an ambient ionization technique for mass spectrometry developed a decade ago to enable rapid and sensitive analyses with little or no sample preparation [1]. DART uses metastable gas atoms (e.g., helium) or molecules that impinge on a surface causing desorption and ionization of the sample. Chemical forensic applications have been a rapidly growing area taking advantage of new methods, including DART, capable of analyzing directly from surfaces at ambient pressure [2]. The technique also is attractive for homeland security purposes, where accurate detection of chemical warfare agents is critical to make informed decisions [3]. High resolution and accurate mass measurement are generally required for application of the technique to real-life samples [4]. Mass measurement accuracy (MMA) is particularly stringent for the unequivocal confirmation of the presence of organophosphorus compounds containing sulfur (S) such as V-series nerve agents [5], which has not been achieved in earlier works documenting inadequate MMA in this regard for several chemicals through the use of time-of-flight (TOF) [4] or even Orbitrap mass analyzers [6]. In addition, application of tandem mass spectrometry (MS/MS) at ultrahigh resolution made possible by Fourier transform mass spectrometry (FTMS) has not been exploited using DART.

With focus on homeland security application, we report here the evaluation of Fourier transform ion cyclotron resonance (FT-ICR) mass spectrometry to detect sulfur-containing small molecules at high resolution to achieve adequate MMA using both DART–MS and DART–MS/MS techniques. When testing fit-for-purpose in a routine experimental setting, chemical warfare agents are commonly mimicked by an analog compound that is safe to study without taking extreme risk [7,8]. Therefore, we also selected an organothiophosphate as a simulant for the reported experiments starting with sample collection in the field and, once the samples had been transported to the laboratory, finishing with DART–FT-ICR analyses.

## 2. Results

**Figure 1:**
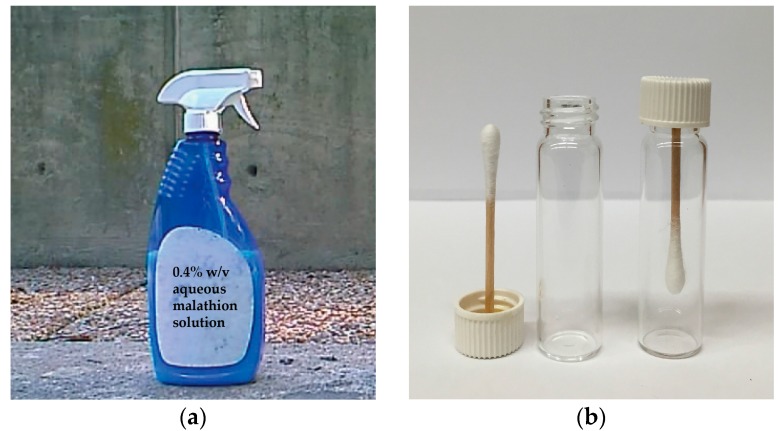
Illustration of the sampling method: (**a**) About 1.3 ± 0.1 mL of aqueous 0.4% *w*/*v* malathion solution was sprayed from approximately 30 cm distance from a household spray bottle (foreground) by a single application of the lever onto a concrete wall in the background; (**b**) After swabbing the surface across the sprayed area using cotton-tipped applicators with their wooden handle glued in scintillation-vial caps (on the **left**), the swabs were screwed into the vials (on the **right**) to protect from sample loss and cross-contamination.

The organothiophosphate chosen for our experiments was diethyl 2-[(dimethoxy- phosphorothioyl)sulfanyl]butanedioate, an insecticide known as malathion. About 1.3 ± 0.1 mL aqueous 0.4% *w*/*v* solution was sprayed from approximately 30 cm distance onto a concrete wall from a household spray bottle by a single application of the lever (Figure 1A). After 30-min air-drying, the sprayed area (a circular pattern of 30 ± 5 cm in diameter, as determined in a separate experiment spraying an aqueous red food color solution from the bottle) was swabbed across using cotton-tipped applicators making sure that repeated swabbing did not run over paths already sampled. By dipping cotton tips into the food color solution and measuring the widths of marking made on paper, we estimated that each swabbing sampled about 9 ± 3 cm^2^ area. Considering sampling efficiency to be a few percent, the cotton tips could pick up the residues from high ng to low µg quantities for subsequent analyses. To protect the collected samples during shipment, the applicators were glued onto scintillation-vial caps and screwed into their vials (Figure 1B). The swabbed samples were transported to the laboratory, so that analyses started 45–60 min after collection in the field. The instrument was tuned and calibrated about an hour before starting the measurements.

**Figure 2:**
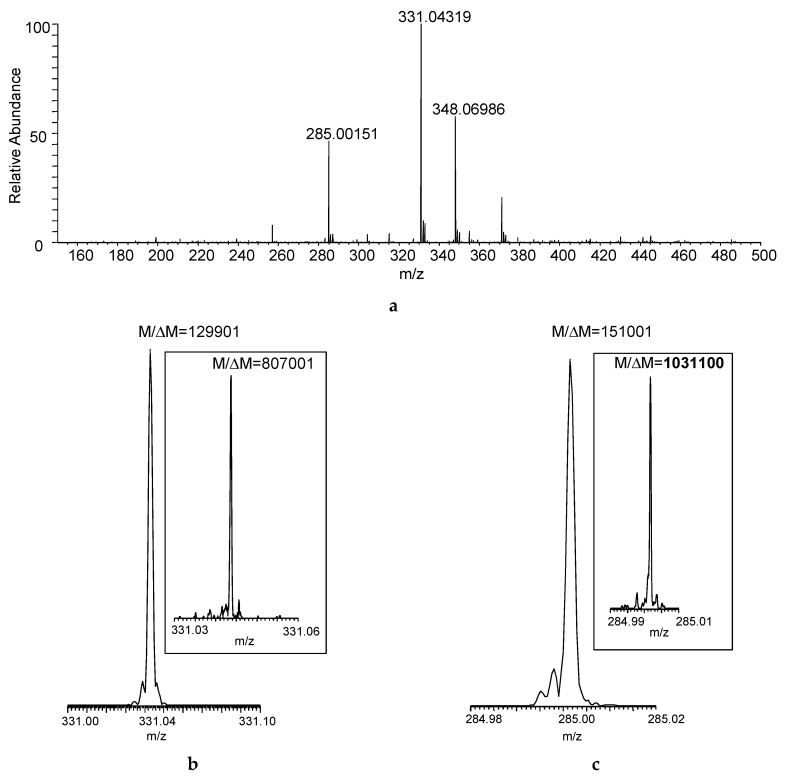
(**a**) Direct analysis in real time (DART) mass spectrum of a sample collected by swabbing from the field (Figure 1) using FT-ICR with desired *M*/Δ*M* set to 100,000 FWHM at *m/z* 400 (Mass spectrum averaged for the entire acquisition period); actual *M*/Δ*M* (FWHM) achieved for (**b**) the protonated malathion (nominal *m/z* 331) and (**c**) DART fragment ion of the compound (nominal *m/z* 285) at set mass resolution of 100,000 at *m/z* 400 (The insets show the actual *M*/Δ*M* with desired mass resolution set to 500,000 at *m/z* 400).

Figure 2a shows the DART–MS recorded at desired resolving power of 100,000 (*M*/Δ*M*, defined at *m*/*z* 400 based on full width at half maximum for the peak and abbreviated as FWHM). Desorption and ionization of the sample directly from the cotton swab resulted in the acquisition of seven mass spectra that reached at least 10% in relative intensity considering the maximum total ion current recorded during the acquisition as a base. The actual resolution shown for the protonated malathion ([M + H]^+^, nominal *m/z* 331) was larger than the set *M*/Δ*M* of 100,000, and the desired resolving power of 500,000 (at *m/z* 400) yielded *M*/Δ*M* > 800,000 (Figure 2b)—albeit obviously at the expense of the number of mass spectra acquired—but the increased *M*/Δ*M* apparently helped in resolving isobaric interferences from the swabbed sample matrix (manifesting as “side-peaks”). With the latter resolution setting, *M*/Δ*M* exceeded 1,000,000 for a DART fragment of the compound (*m/z* 285, Figure 2c).

**Figure A1:**
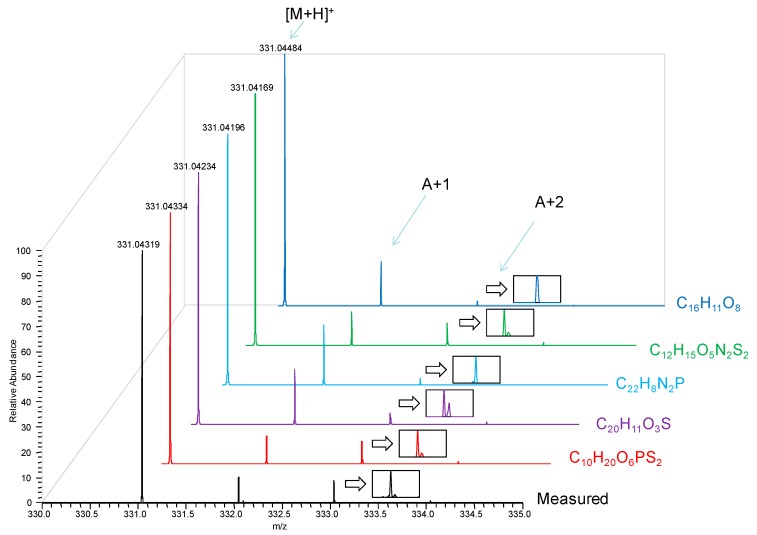
Relative abundances of the measured A+1 and A+2 isotope peaks (bottom trace), as well as isotopic fine structure of the A+2 isotope peak (inset over the bottom trace labeled “Measured”) for the protonated analyte also affords the best match with those predicted for C_10_H_20_O_6_PS_2_. The isotope peak abundances and isotopic fine structures for the indicated elemental compositions listed in Table 1**a** (colored traces and insets, respectively) were predicted presuming *M*/Δ*M* of 120,000, FWHM.

Accurate masses for the three most intense ions detected in mass spectrum and matched to formulae with MMA of less than 5 ppm (commonly considered the maximum accuracy reliably reached by TOF analyzers using internal mass calibration or “lock mass” [9,10]) were listed in Table 1. There was no ambiguity regarding the mechanism of ionization, and all ions in our DART mass spectra appeared to form by proton or cation addition to the analyte, as well as neutral loss from these primary ions. Therefore, we limited formula search from the measured accurate masses to even-electron ions. Nevertheless, MMA of <1 ppm was required for unequivocal matching to the correct formulae. In addition to verifying the [M + H]^+^ (protonated malathion) through its accurate mass, the ion at nominal *m/z* 348 was found to be [M + NH_4_]^+^ from the adduction of ammonium ion, while the ion at nominal *m/z* 285 originated from the loss of C_2_H_6_O (ethanol) possibly from the [M + H]^+^ of the analyte. The correct molecular formulae were also confirmed through the analysis of the isotopic peaks and matching to their predicted abundances and isotopic fine structures (see Figure A1 in the Appendix as an example).

Table 1Formulae and MMA calculated from accurate masses for the three most intense ions of the DART–MS recording made by FT-ICR, with *M*/Δ*M* of 100,000 FWHM at *m/z* 400 and using external mass calibration. The search range for formulae was C_0–25_H_0–50_O_0–8_N_0–2_P_0–1_S_0–2_, and the output was limited to even-electron ions.

**Table:** (**a**) Formulae with ≤5 ppm MMA for *m/z* 331.04319.

Rank	Formula	MMA (ppm)
1	C_10_H_20_O_6_PS_2_	−0.46
2	C_20_H_11_O_3_S	2.56
3	C_22_H_8_N_2_P	3.71
4	C_12_H_15_O_5_N_2_S_2_	4.53
5	C_16_H_11_O_8_	5.00

**Table:** (**b**) Formulae with ≤5 ppm MMA for *m/z* 348.06986.

Rank	Formula	MMA (ppm)
1	C_10_H_23_O_6_NPS_2_	−0.09
2	C_20_H_14_O_3_NS	2.79
3	C_16_H_14_O_8_N	−4.40

**Table:** (**c**) Formulae with ≤5 ppm MMA for *m/z* 285.00151.

Rank	Formula	MMA (ppm)
1	C_8_H_14_O_5_PS_2_	0.113
2	C_18_H_5_O_2_S	2.63

**Figure 3:**
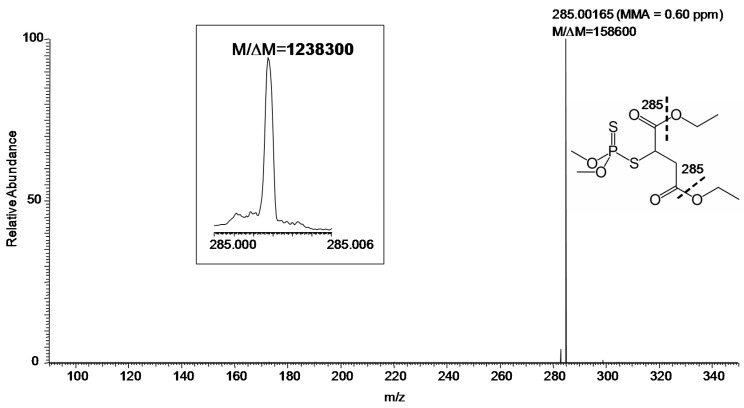
Product ion MS/MS of protonated malathion (*m/z* 331) from a sample collected by swabbing from the field (Figure 1) followed by DART–MS using a hybrid mass spectrometer with CID in the linear ion trap and fragment ions detected in the FT-ICR (set *M*/Δ*M* of 100,000 FWHM at *m/z* 400). Inset: The fragment ion *m/z* 285 (set *M*/Δ*M* of 500,000 FWHM at *m/z* 400). The fragmentation leading to the observed ion is indicated by the thick dashed line on the displayed structure of the compound.

MS/MS (especially at FTMS resolution and MMA) adds another level of certainty to identification. Using the hybrid instrument available for our study, collision-induced dissociation (CID) can be efficiently carried out in the linear ion trap [11] with the fragment ions transferred to the FT-ICR for measurement [12]. The CID product ion spectrum of *m/z* 331 with desired resolution of 100,000 (at *m/z* 400) was shown in Figure 3. Specifically, the measurements gave *M*/Δ*M* and MMA of 158,600 and 0.60 ppm, respectively, for the major fragment ion (*m/z* 285) of protonated malathion. Again, *M*/Δ*M* exceeded 1,000,000 for this fragment ion, when the desired resolution at *m/z* 400 was set to 500,000, as displayed in the inset of Figure 3.

## 3. Discussion

With growing threats of terrorist attacks involving chemical and biological weapons [13], targeted countries have been striving to enable rapid detection and accurate identification of agents that could be deployed, so that informed decisions are made based on the obtained results and without much delay. Ambient mass spectrometry that involves little or no sample preparation such as DART offers a method to address the challenges regarding these applications [3]. However, sulfur-containing agents such as the V-series of nerve agents [5,14] have been demanding with regard to system requirements, and high-resolution/high-MMA data acquisitions exceeding the performance of TOF analyzers [9,10] are needed. Sulfur is among the atoms that can be counted in a compound through analysis of the isotope peaks and isotopic fine structures [15], albeit that the practical value of sulfur-counting lies in mass spectrometry of proteins [16]. Therefore, our work focused on the evaluation of MMA using malathion as an organothiophosphate test compound.

Our study also mimicked actual sampling from the environment (“field”). A surface swabbing technique has been employed for rapid sample collection to DART–MS analyses [6,17]. Therefore, we also adopted this method for our studies. The use of FTMS has well addressed the challenges involved in the analysis of the organothiophosphate test agent. Using DART–FT-ICR mass spectrometry and MS/MS in a hybrid instrument, *M*/Δ*M* exceeding (for the first time with this method of desorption and ionization) 1,000,000 FWHM was documented for ions attributable to the analyte—in particular, for a product ion in the full-scan CID-MS/MS spectrum of the protonated compound. MMA below 1 ppm was obtained without internal reference (“lock mass”) and, thus, relying merely upon an external mass calibration for all detected species that belonged to the test compound. Therefore, internal mass calibration that might rely on, for example, common organic contaminants and require another step in raw data processing [18] would be unnecessary. Variation of the ionization mechanisms in the DART source [19] may be a factor to consider upon obtaining the molecular formulae from the measured accurate masses, although such ambiguity was not observed in our study (*i.e*., only even-electron ions were identified in the recorded mass spectra, and odd-electron ions due to Penning ionization were absent). For the chosen organothiophosphate, about 75% of the analyte was detected as intact molecular ions ([M + H]^+^ and [M + NH_4_]^+^) in the DART mass spectrum. The relatively small extent of in-source fragmentation (the loss of ethanol from the protonated molecule) did not interfere with the analyses performed. Fragmentation is controlled mainly by the voltages at electrodes of the DART source and by the voltage at the inlet of mass spectrometer [20]; therefore, these voltages may be optimized, if necessary, to preserve intact molecular ions for subsequent mass spectrometric and MS/MS analyses. Alternatively, in-source fragmentation may also be induced through increasing the electrode or inlet voltages to distinguish fragment ions from intact species.

It is plausible that the reported results could be matched by newer, non-ICR-based FTMS instruments (Orbitraps), although attention should be given to detailed inspection and manual tuning of the installation parameters to reach sub-ppm MMA upon their use [16]. In conclusion, this high MMA, currently only achievable through FTMS, was required for the unequivocal identification of the molecular formulae from the ions of the organothiophosphate analyte after sample collection by swabbing, followed by DART–MS and MS/MS analyses.

## 4. Materials and Methods

Malathion concentrate (Ortho^®^ Malathion Plus™, The Ortho Group, Marysville, OH, USA), Red Food Color Solution containing FD&C Reds 40 and 3 (McCormick, Hunt Valley, MD, USA), methyl cyanoacrylate (Super Glue Corp., Ontario, CA, USA) and spray bottle were purchased from a local hardware store or supermarket. Tap water was used to prepare tests solutions (0.4% *w*/*v* concentration for malathion and 1:100 *v*/*v* dilution from the red food color solution). Fisherbrand™ 9.5 mm cotton-tipped applicators with 15 cm × 2.2 mm o.d. wooden handles, Kimble™ 7 mL borosilicate glass scintillation vials and white plastic screw caps with cork backed foil liner (Thermo Scientific, Pittsburgh, PA, USA) were used to make the simple swabbing tool shown in Figure 1. The wooden handle of the applicators were cut to 4.5 cm and, after stripping off the aluminum foil, glued in the middle of the liners using methyl cyanoacrylate. Marks on a printer paper made by the cotton tips dipped into the diluted red food color solution were measured after drying and using an Ultratest^®^ 0–7in Vernier caliper (General Tools, Secaucus, NJ, USA). These and other coarse measurements reported in the beginning of the Results section were done in triplicate.

The DART source was a model 100 device (IonSense, Saugus, MA, USA) operated with helium as working gas at a flow rate of 12 L/min and temperature of 300 °C. The discharge needle voltage was set to 3800 V and the voltages of electrode 1 and electrode 2 to +400 V and +500 V, respectively. The mass spectrometer was a LTQ-FT hybrid instrument (Thermo Fisher Scientific, Bremen, Germany) combining a linear ion trap (linear trapping quadrupole, LTQ) with a 7-Tesla FT-ICR through a series of transfer octapoles [12]. External mass calibration was performed according to the manufacturer’s specifications and using electrospray ionization with the LTQ-FT Calibration Solution (caffeine, the peptide Met-Arg-Phe-Ala, and Ultramark 1621) supplied by a syringe pump built into the LTQ unit and fitted with a 500-µL syringe (Hamilton, Reno, NV, USA). The acquisitions were performed under automatic gain control to manage the ion population in the instrument and avoid space-charge effects [21]. Based on the ion flux estimated from a single prescan by the LTQ (usually lasting <5 ms with 100 ms allowed as maximum ion injection time), trapping times for analytical scans in FT-ICR cell were automatically adjusted for 10^6^ and 10^5^ as target counts to acquire full-scan MS and MS/MS spectra, respectively. CID was done in the LTQ using 1.0 Th precursor isolation width, 30% relative collision energy and 30-ms activation time. Full-scan MS and MS/MS spectra in the FT-ICR were acquired from *m/z* 150 to 500 and *m/z* 90 to 350 with resolving powers at *m/z* 400 set to 100,000 and 500,000, respectively. The instrument was controlled through the manufacturer’s XCalibur (version 2.0) and TunePlus (version 2.2) software. Spectra were displayed by the Qual Browser program of XCalibur, which was also used for calculating formulae from the measured accurate masses.

## Abbreviations

CID: collision-induced dissociation
DART: direct analysis in real time
FT-ICR: Fourier transform ion cyclotron resonance
FTMS: Fourier transform mass spectrometry
FWHM: full width at half maximum
LTQ: linear trapping quadrupole
*M*/Δ*M*: mass resolution
MMA: mass measurement accuracy
MS: mass spectrometry
MS/MS: tandem mass spectrometry
TOF: time-of-flight
